# Nep1-like proteins as a target for plant pathogen control

**DOI:** 10.1371/journal.ppat.1009477

**Published:** 2021-04-15

**Authors:** Katja Pirc, Vesna Hodnik, Tina Snoj, Tea Lenarčič, Simon Caserman, Marjetka Podobnik, Hannah Böhm, Isabell Albert, Anita Kotar, Janez Plavec, Jure Borišek, Martina Damuzzo, Alessandra Magistrato, Boris Brus, Izidor Sosič, Stanislav Gobec, Thorsten Nürnberger, Gregor Anderluh

**Affiliations:** 1 Department of Molecular Biology and Nanobiotechnology, National Institute of Chemistry, Ljubljana, Slovenia; 2 Department of Biology, Biotechnical Faculty, University of Ljubljana, Ljubljana, Slovenia; 3 Center of Plant Molecular Biology (ZMBP), Eberhard-Karls-University Tübingen, Tübingen, Germany; 4 Slovenian NMR Center, National Institute of Chemistry, Ljubljana, Slovenia; 5 Theory Department, National Institute of Chemistry, Ljubljana, Slovenia; 6 CNR-IOM-Democritos at International School for Advanced Studies (SISSA), Trieste, Italy; 7 Faculty of Pharmacy, University of Ljubljana, Ljubljana, Slovenia; 8 Department of Biochemistry, University of Johannesburg, Auckland Park, Johannesburg, South Africa; Nanjing Agricultural University, CHINA

## Abstract

The lack of efficient methods to control the major diseases of crops most important to agriculture leads to huge economic losses and seriously threatens global food security. Many of the most important microbial plant pathogens, including bacteria, fungi, and oomycetes, secrete necrosis- and ethylene-inducing peptide 1 (Nep1)-like proteins (NLPs), which critically contribute to the virulence and spread of the disease. NLPs are cytotoxic to eudicot plants, as they disturb the plant plasma membrane by binding to specific plant membrane sphingolipid receptors. Their pivotal role in plant infection and broad taxonomic distribution makes NLPs a promising target for the development of novel phytopharmaceutical compounds. To identify compounds that bind to NLPs from the oomycetes *Pythium aphanidermatum* and *Phytophthora parasitica*, a library of 587 small molecules, most of which are commercially unavailable, was screened by surface plasmon resonance. Importantly, compounds that exhibited the highest affinity to NLPs were also found to inhibit NLP-mediated necrosis in tobacco leaves and *Phytophthora infestans* growth on potato leaves. Saturation transfer difference-nuclear magnetic resonance and molecular modelling of the most promising compound, anthranilic acid derivative, confirmed stable binding to the NLP protein, which resulted in decreased necrotic activity and reduced ion leakage from tobacco leaves. We, therefore, confirmed that NLPs are an appealing target for the development of novel phytopharmaceutical agents and strategies, which aim to directly interfere with the function of these major microbial virulence factors. The compounds identified in this study represent lead structures for further optimization and antimicrobial product development.

## Author summary

Nep1-like proteins (NLPs) constitute a large protein family of virulent agents that are prevalent in different microbial taxa such as bacteria, oomycetes, and fungi. NLPs represent an important molecular target for the development of novel plant protection products due to their crucial role in plant diseases and their presence in a number of different organisms. We have identified three small molecular weight compounds that bind to and inhibit the cytotoxic activity of NLPs. These compounds also inhibited NLP-mediated necrosis in tobacco leaves and the growth of the important plant pathogen *Phytophthora infestans* on potato leaves.

## Introduction

Plant pathogens cause diverse diseases that affect crop yield and food quality, which leads to extensive annual financial losses worldwide. Thus, crops can be severely affected in the absence of pest control. For example, approximately 50% of wheat and more than 80% of cotton production would be eliminated by different diseases in the absence of agrochemical interventions [1]. Controlling plant pathogens is thus of crucial importance for modern agriculture. Fungicides are widely used in developed agricultural production to control diseases and maintain sufficient crop yield and product quality. However, the mechanism of action is not known for most fungicides, and there may be possible side effects for the host plants. The use of nonspecific chemical strategies to control crop production can also have deleterious effects on the environment and human health [2]. New compounds and strategies with better effectiveness, lower application dosage, higher selectivity, and fewer costs and lower environmental impact are thus highly desirable.

Pathogens have evolved a plethora of effectors, i.e., proteins and small molecules, to manipulate the cellular processes of hosts and establish parasitic relationships [3,4]. Secreted effector molecules include necrosis- and ethylene-inducing peptide 1 (Nep1)-like proteins (NLPs), which constitute one of the largest microbial protein families with more than 1,700 identified homologues [5]. NLPs are widely distributed among prokaryotic and eukaryotic microorganisms (i.e., bacteria, fungi, and oomycetes) and have been shown to aid the infection of eudicot host plants [6]. Such pathogens may infect a wide range of different crops, including potato, tomato, soybean, grapevine, and tobacco. NLPs exhibit two main actions in plant-pathogen interactions, as they can (i) act as toxin-like virulence factors that induce tissue necrosis and (ii) trigger plant immune responses [7]. Plant responses to NLPs are reminiscent of pattern-triggered plant immunity comprising the biosynthesis of ethylene, production of reactive oxygen species, and production and release of antimicrobial compounds [8,9]. The immunogenic activities of NLPs are either mediated by the pattern recognition receptor RLP23 in *Arabidopsis* and related Brassicaceae species or are triggered by the deleterious impact of toxic NLPs on eudicot plant host membranes [10]. Non-cytotoxic members of the NLP family have also been reported, indicating that NLPs have undergone functional diversification, including functions beyond host infection [11–13]. Many NLPs are expressed by necrotrophic or hemibiotrophic plant pathogens at the onset of host infection or during the transition from biotrophic to necrotrophic growth [5]. The number of NLPs encoded by individual microbial species varies considerably, with an expansion of *NLP* genes in the genomes of oomycetes, suggesting an important role of these proteins in the life cycle of this pathogen [5]. NLPs have been described in several different species of the genus *Phytophthora*: *P*. *infestans* [14], *P*. *parasitica* [15], *P*. *capsici* [16], *P*. *megakarya* [17], *P*. *ramorum* [14], and *P*. *sojae* [18]. Furthermore, NLPs exhibit an unusual host selectivity, as they only elicit diverse defence reactions and cell death in eudicots but not monocots [19]. It has been shown that NLPs function as cytolytic toxins that disrupt the plasma membrane integrity of eudicots, thereby causing cytotoxicity [20]. Recently, the structural basis for this unusual host specificity has been resolved and attributed to different structural features between eudicot and monocot plasma membrane glycosylinositol phosphorylceramides (GIPCs), which act as target receptors for toxins [21].

As NLPs play a crucial role in plant infection, they represent a promising target for the development of new phytoprotective substances that could prevent the devastating effects of microbial plant pathogens that produce these cytolysins. The widespread presence of structurally conserved NLPs in plant-associated bacteria, fungi, and oomycetes indicates that a single potential inhibitor could protect against a wide variety of pathogenic microorganisms. Herein, we employed a surface plasmon resonance (SPR)-based approach to identify small molecular weight NLP binders that exhibit inhibitory effects on the necrotic activities of NLPs. We have identified three molecules that bind to NLPs in the micromolar range and reduce NLP-induced necrosis in tobacco leaves. Importantly, two putative inhibitors also reduced *Phytophthora* growth on potato plants. Nuclear Magnetic Resonance (NMR) and all-atoms molecular dynamics simulations supplied structural information for the binding of these inhibitors to NLP. As a result, NLPs appear as promising targets for the further development of novel phytopharmaceutical compounds and plant protection strategies.

## Results

### Binding analysis

**Fig 1:**
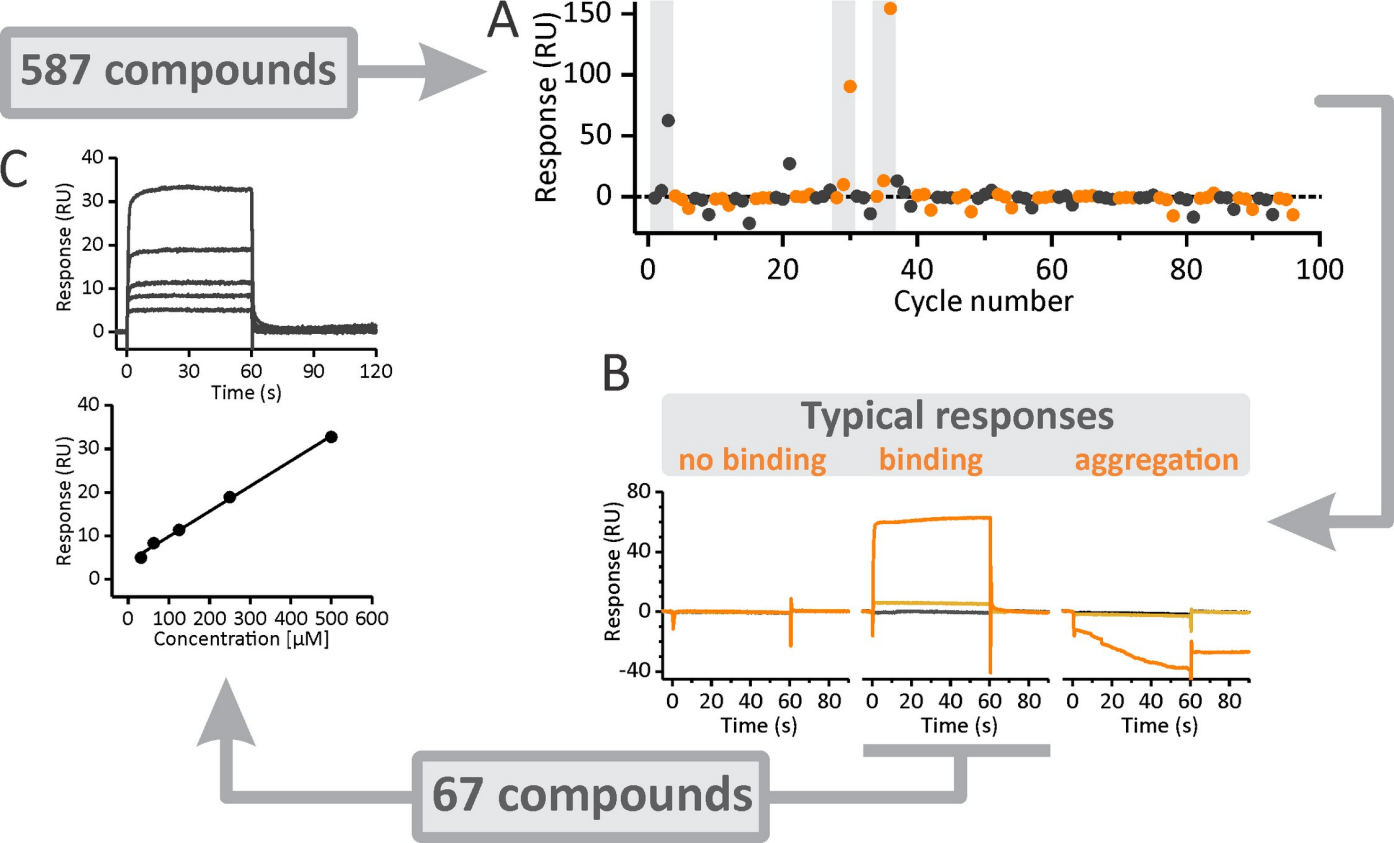
Surface plasmon resonance-based screening of compounds. (A) The binding of each compound to NLP_Pya_ was initially tested at two concentrations, 20 and 200 μM. A buffer control was injected in between different compounds. The dots represent binding responses 5 s before the end of each injection. (B) Sensorgrams were used to obtain the data in (A) and are presented for the buffer injection (black), 20 μM compound (light orange), and 200 μM compound (dark orange). Typical responses included no binding at any concentration (left), aggregation of compounds at the higher concentration (right), or concentration-dependent binding (middle). (C) Afterwards, 67 compounds that exhibited concentration-dependent binding to NLP_Pya_ were further screened at an extended range of concentrations. The titrations shown are for the compound **5D11** [61], which was titrated with concentrations ranging from 31 to 500 μM (from the bottom to the top in the upper graph). **5D11** does not exhibit saturated binding for the tested concentration range (bottom graph). The line is a linear fit to the responses obtained from the sensorgrams presented in the upper panel (5 s before the end of the injection).

A total of 587 chemical compounds were selected according to particular structural and physicochemical characteristics that enable potential further phytopharmaceutical drug development [22]. Approximately one-third of these compounds have molecular weights of <300 Da, indicating their fragment-like nature and suitability for potential optimization. Initial screening for potential NLP binders included sequential injections of all compounds at two concentrations over the sensor-chip-immobilized NLP from *P*. *aphanidermatum* (NLP_Pya_) (Fig 1A), as NLP_Pya_ is becoming an important model for studying NLP interactions with lipid membranes [20,21]. The different compounds exhibited typical SPR responses that suggested either the absence of binding, analyte aggregation at higher concentrations, or concentration-dependent binding (Fig 1B). Compounds that were only partially soluble in the SPR running buffer or did not bind were omitted from the subsequent tests. To select promising binders, a binding point was assigned to each sensorgram, i.e., the response 5 s prior to the end of injection (Fig 1A). Concentration-dependent responses were observed for 67 compounds (highlighted by gray bars in Fig 1A), which were chosen for further detailed binding experiments (Fig 1C). The subsequent titrations revealed binding of many compounds to the protein; however, the interactions were weak and did not reach saturation in the tested concentration ranges, as shown in Fig 1C for one of the compounds. Typically, compound concentrations of up to 0.5 mM were assessed, as many of the compounds could not be solubilized in the SPR running buffer at higher concentrations.

**Fig 2:**
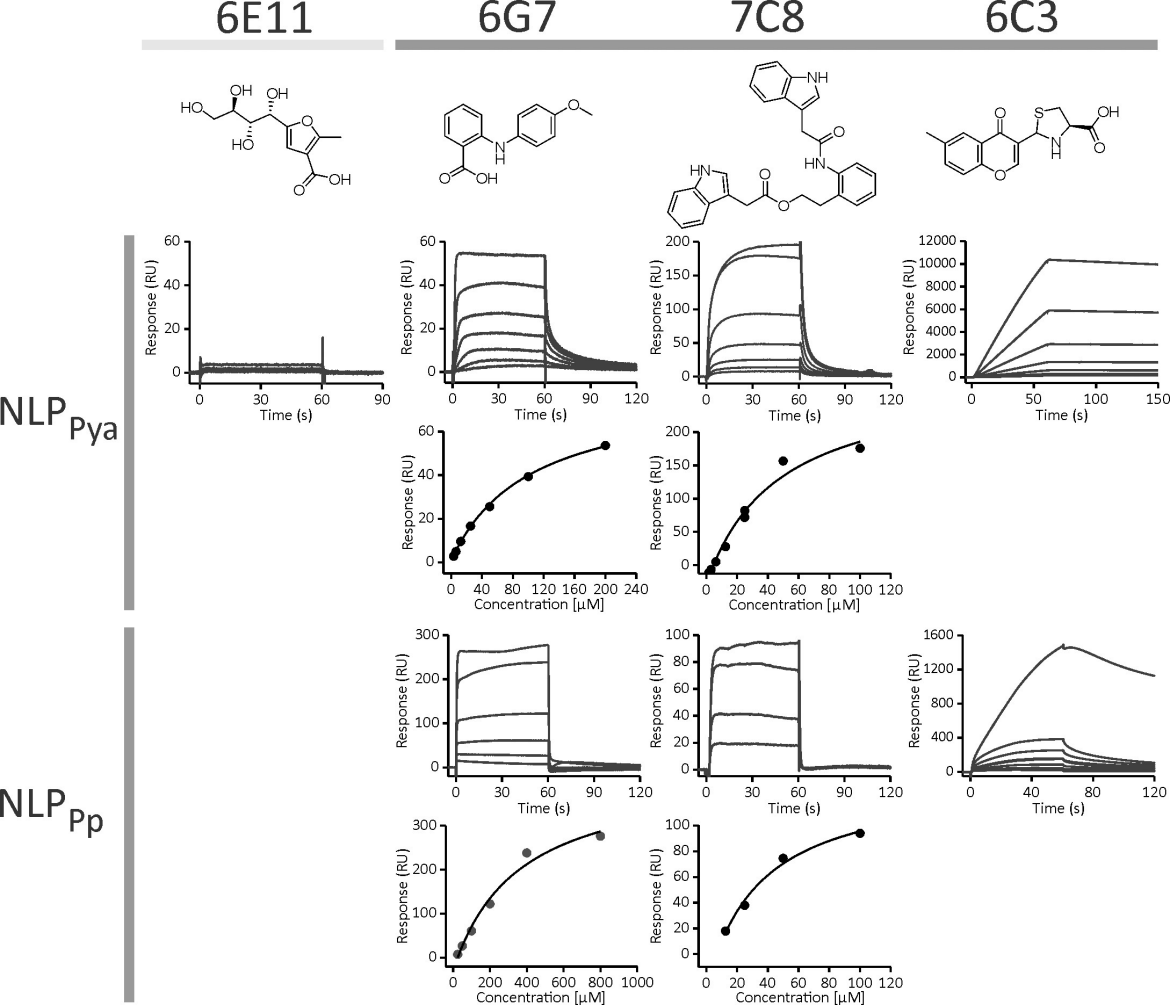
Binding of compounds 6G7, 7C8, and 6C3 to NLP_Pya_ and NLP_Pp_. The most promising binders were compounds **6G7** and **7C8**. The binding response of **6C3** exceeded the expected response for a 1:1 interaction, indicating multi-site binding. Compound **6E11** was used as a control. Data were fit to the steady-state affinity model.

The most promising NLP_Pya_ binders were compounds **6G7** and **7C8** with K_D_ values of 130.9 ± 36.4 μM (n = 6) and 52.8 ± 6.0 μM (n = 3), respectively (Fig 2). **6C3** exhibited stable binding to NLP_Pya_; however, the maximum binding response was at least 100 times higher than expected for 1:1 binding, which is indicative of a promiscuous inhibitor [23]. **6E11** did not bind to NLP_Pya_ (Fig 2) and was thus selected as a negative control for all subsequent experiments. The binding of **6C3**, **6G7**, and **7C8** was additionally tested on another NLP, NLP_Pp_, which is secreted by *P*. *parasitica* [15]. Similarly, **6C3** exhibited nonspecific binding, and **6G7** and **7C8** exhibited K_D_ values of 71.9 ± 19.1 μM (n = 2) and 40.5 ± 29.7 μM (n = 4), respectively (Fig 2).

### The selected compounds inhibited NLP-induced necrosis

The toxic effects of NLPs on plant tissues are primarily observed as tissue necrosis [19,24], a gradual decay of leaf mesophyll cells and chlorosis of the leaves. The NLP-induced leaf tissue necrosis provides a robust and efficient functional assay for the identification of NLP inhibitors. Infiltration assays on tobacco leaves were used to test the potential inhibitory activity of **6G7**, **6C3**, and **7C8** (S1 Fig), which exhibited the highest affinity to NLPs in the SPR screening experiments (Fig 2). The necrotic lesions were inspected after 24 h. The compounds alone (at 1 mM) did not exert toxic effects on leaf tissues (S1A Fig). Next, we injected 400 nM NLP_Pp_ and 200 nM NLP_Pya_ alone or together with increasing concentrations of compounds subaxially into the leaf (S1 Fig). **6E11**, which did not interact with NLP_Pya_ in the SPR assay (Fig 2), did not affect NLP_Pp_ cytotoxicity (S1B Fig). All three compounds that bound to NLPs (Fig 2) inhibited NLP_Pp_- and NLP_Pya_-induced necrosis (S1B and S1C Fig).

### The selected compounds inhibited the growth of *P*. *infestans*

**Fig 3:**
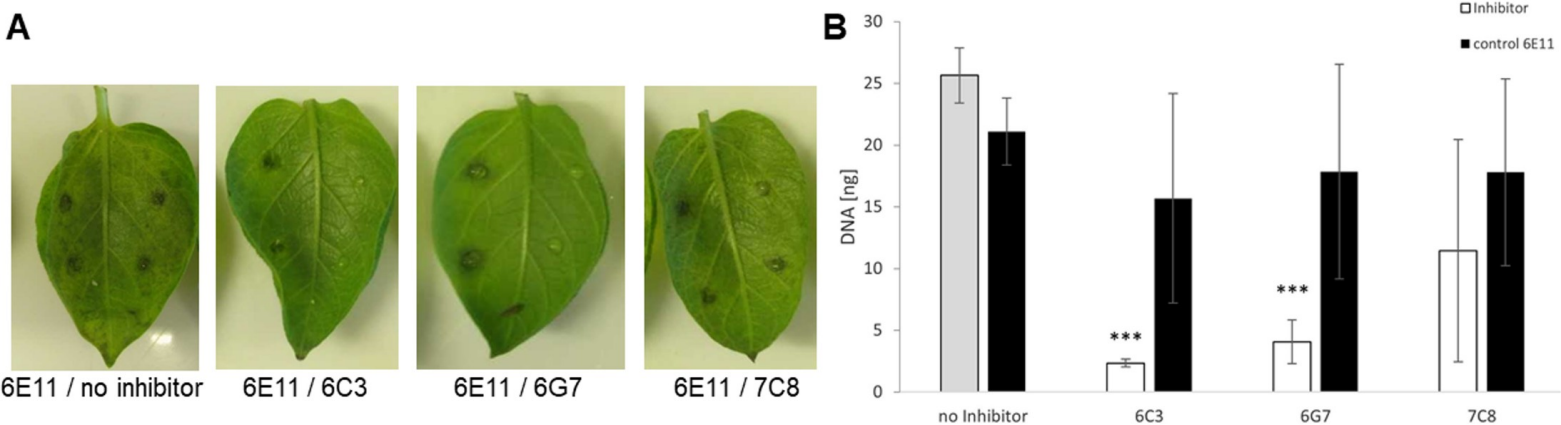
The inhibitory effects of the compounds on the growth of the pathogen *Phytophthora infestans*. (A) *P*. *infestans* growth on potato leaves (*Solanum tuberosum*). Each potato leaf was inoculated with both 1 mM of the control compound 6E11 (left half of the leaf) and 1 mM of the test compound (right half of the leaf). The leaves are representatives of ten repetitions, each conducted in parallel (applied on the top and the bottom of the leaf). (B) Quantification of *P*. *infestans* growth on potato leaves 4 days after infection by real-time PCR. ***P<0.001 between the test and control compound; Student’s t-test.

*P*. *infestans* is one of the most devastating plant pathogens that causes major damage to potato and tomato production worldwide. To assess their possible inhibitory effect, **6C3**, **6G7**, and **7C8** were applied to potato leaves together with spore preparations of *P*. *infestans*. Reductions in the size and coloring of infection spots indicated that the infections were largely reduced or disappeared in the presence of **6C3**, **6G7**, and **7C8**, but not in the presence of **6E11** (Fig 3A). Similarly, RT-PCR revealed that pathogen growth was significantly reduced in the presence of **6C3**, **6G7**, and **7C8** (Fig 3B).

Phytopharmaceuticals are only useful if they are not toxic to humans and other living organisms. Therefore, the toxicities of **6G7**, **6C3**, and **7C8** were tested on the human colon epithelial adenocarcinoma cell line Caco-2 (S2 Fig). Cells were incubated overnight with the selected compounds at different concentrations, and cell toxicity was monitored with an MTT assay (S1 Methods). The lowest tested concentration of **7C8** (3.125 μM) reduced Caco-2 cell viability by 35%, and 12.5 μM **7C8** reduced cell viability to 20% of the untreated control. **6C3** did not affect cell viability at concentrations below 500 μM, while concentrations of 1 mM and 2 mM reduced cell viability by 8% and 15%, respectively. **6G7** did not affect cell viability, even at concentrations approximately 10 times higher than its K_D_ for NLPs.

### The interaction of 6G7 with NLP_Pya_ as determined by STD-NMR

The most promising candidate for further biophysical and functional evaluation was **6G7** according to its functional properties, solubility, and SPR results. We encountered solubility problems with **7C8**, which was poorly soluble at concentrations of >180 μM using 5% DMSO, while **6C3** exhibited promiscuous binding in SPR experiments (Fig 2), which is not desirable for development of specific inhibitors. We, therefore, performed in-depth characterization of **6G7** biophysical and functional properties.

**Fig 4:**
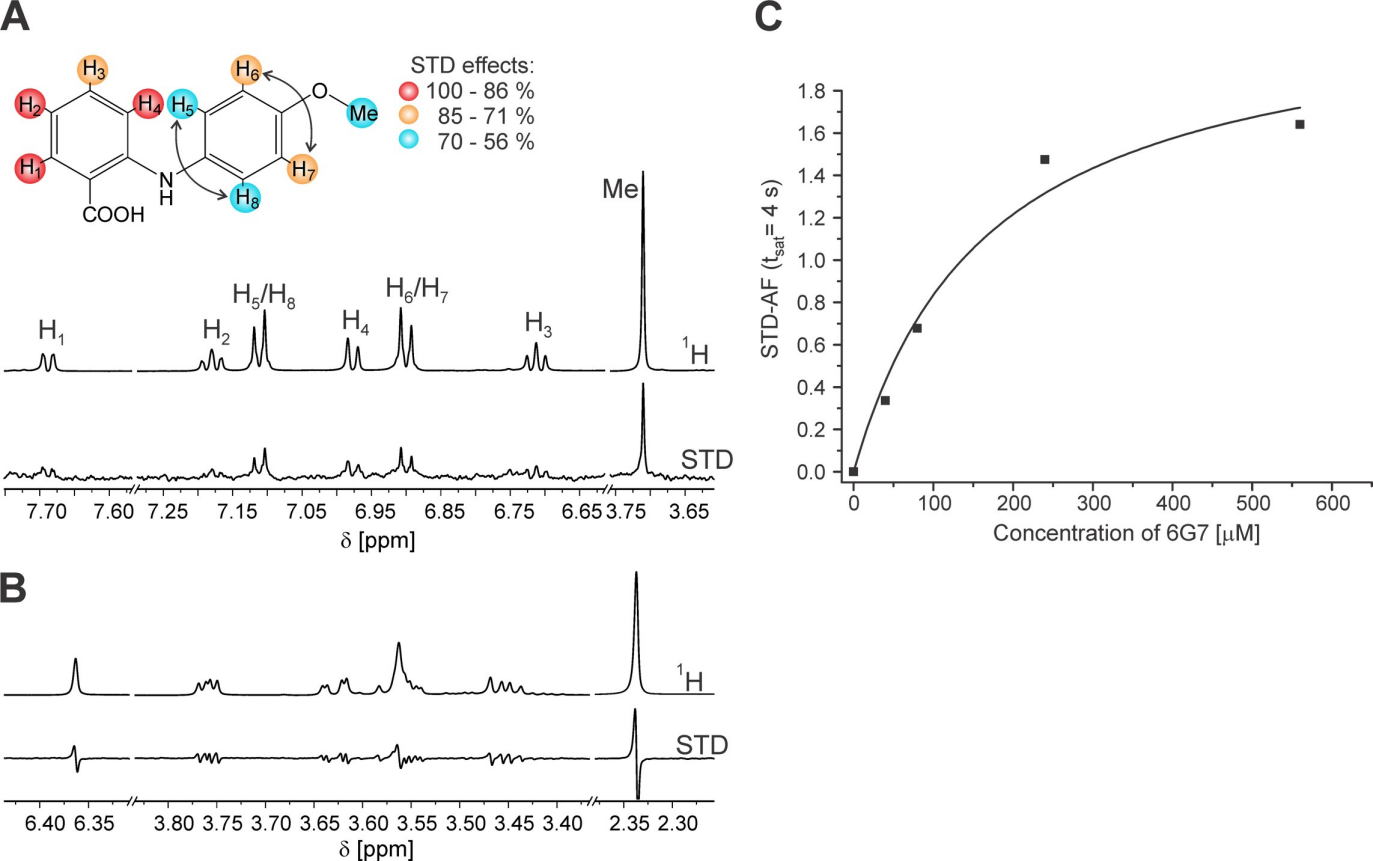
Saturation transfer difference-nuclear magnetic resonance (STD-NMR) analysis of 6G7. (A) ^1^H and STD-NMR spectra of ligand **6G7** in the presence of NLP_Pya_. The NMR spectra were recorded at 25°C, 4 s saturation time, 400 μM ligand concentration, and 1:50 receptor-to-ligand ratio in 25 mM Tris-*d*_11_ and 150 mM NaCl in ^2^H_2_O and 5% DMSO-*d*_6_ at 600 MHz. Signals are labeled according to the atom numbers shown in the scheme, in which the relative degree of hydrogen atom saturation is marked with a corresponding color scale normalized to H_1_. Arrows indicate the overall STD effects for the symmetric atoms H_5/8_ and H_6/7_. (B) ^1^H and STD-NMR spectra of **6E11** in the presence of NLP_Pya_ under the same experimental conditions as in (A). (C) STD-amplification factor as a function of **6G7** concentration (0−560 μM). Experimental data were fit to Eq 2.

We independently confirmed its interaction with NLP_Pya_ by using the saturation transfer difference-nuclear magnetic resonance (STD-NMR) approach. We observed clear STD signals in the presence of **6G7** (Fig 4A), but not in the presence of the negative control **6E11** (Fig 4B). Epitope mapping revealed the highest relative STD effects for the **6G7** protons H_1_, H_2_, and H_4_, indicating that their corresponding aromatic ring is located closest to NLP_Pya_ (Fig 4A). In comparison, the other aromatic ring of **6G7** is positioned further away from the surface of NLP_Pya_, as is demonstrated by the lower STD effects of protons H_5_−H_8_. Accordingly, the lowest STD effect was detected for the methyl moiety. STD-NMR experiments at different **6G7** concentrations allowed for the estimation of K_D_ at 150 ± 7 μM (Fig 4C), which is comparable with the value determined by SPR.

### Binding mode assessment by molecular dynamics simulations

**Fig 5:**
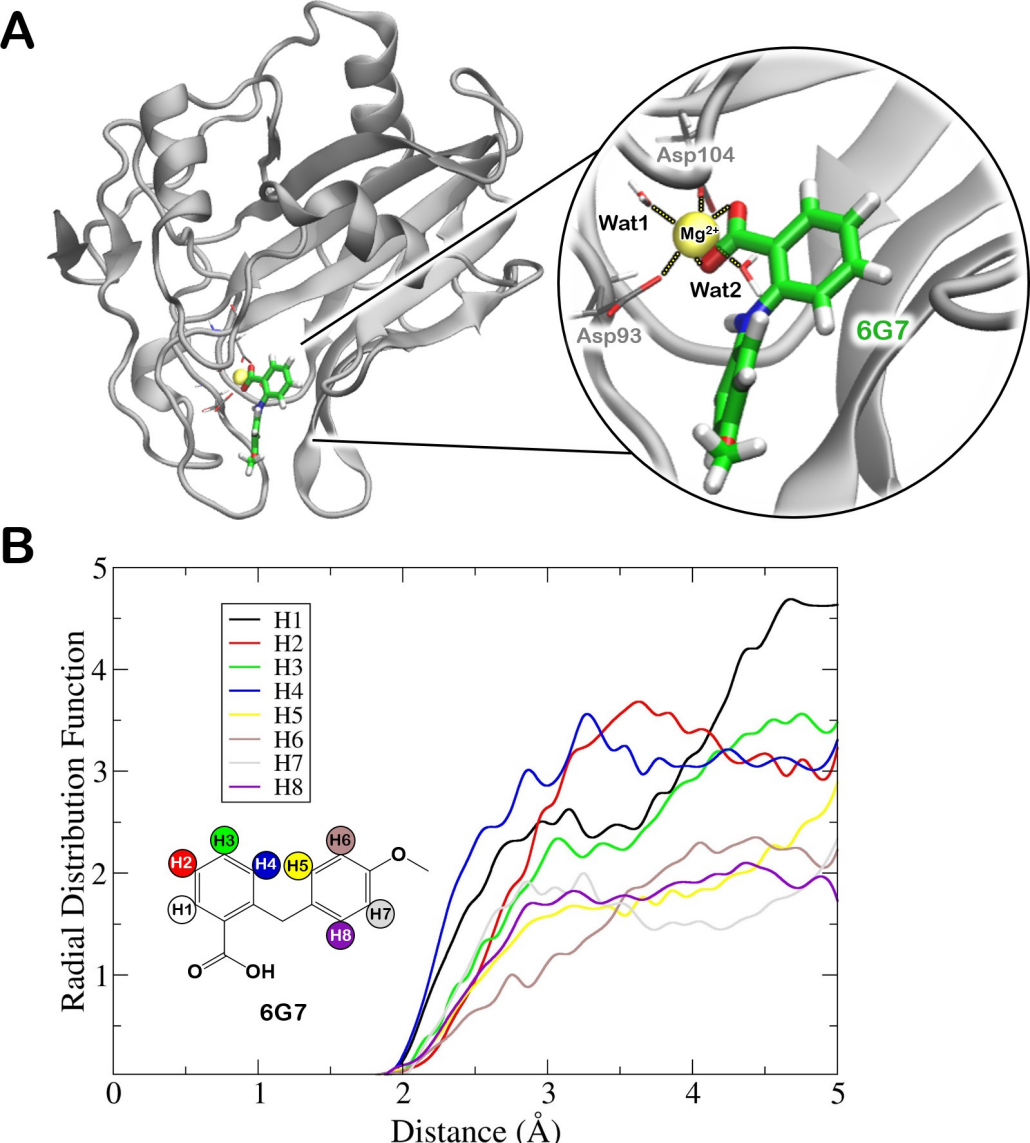
Inhibitor’s binding pose prediction. (A) Complex of NLP_Pya_ with the **6G7** ligand and inset of **6G7** binding mode, as obtained after ~5 ps of quantum/classical (QM/MM) simulations. The **6G7** carboxyl group bound to Mg^2+^ ion, whose octahedral coordination sphere is completed by three water molecules, and by the NLP_Pya_ residues Asp93 and Asp104. The protein is represented as gray cartoons, the Mg^2+^ ion as a yellow van deer Waals sphere, while the residues completing the Mg^2+^ coordination sphere and the ligand are shown in licorice with carbon, oxygen, nitrogen and hydrogen atoms depicted in gray (for protein), green (for inhibitor), red, blue and white, respectively. (B) Radial distribution function plotting the probability density of finding a NLP_Pya_ protein hydrogen atoms at r distance (Å) from any hydrogen atoms of **6G7**.

In order to explore the binding mode of compound **6G7**, we initially attempted at identifying the potential ligand’s binding sites with various small molecule probes [25] on the NLP_Pya_ crystal structure (PDB ID 3GNZ) and two representative structures obtained from a cluster analysis of a μs-long molecular dynamics (MD) simulations trajectory of NLP_Pya_ in explicit solvent [13]. Three binding sites were identified (S3 Fig). Among these the central cavity harbouring the Mg^2+^ ion, implicated in plant membrane sphingolipid receptor recognition [21], was the most likely binding site according to molecular docking simulations [26]. In addition, this was the only cavity where the ligand was retained in subsequent force field MD simulations (S4 Fig). Indeed, **6G7** remained stably bound to this cavity for 1 μs, while it rapidly dissociated from the two other identified binding sites within few ns of MD simulations. Due to the limitations of force fields in the description of Mg^2+^ ions [27] the binding pose of **6G7** was also refined by performing 5 ps of hybrid quantum/classical (QM/MM) MD simulation, where the ligand, the metal and its coordination sphere were treated at QM level of theory. As a result, the carboxyl group of **6G7** coordinated the Mg^2+^ ion, while the Asp93 and Asp104 residues of NLP_Pya_ and two water molecules completed the octahedral coordination sphere of the metal (Fig 5A). In order to assess the agreement of this binding pose with the STD-NMR experiments, we calculated the radial distribution function of **6G7** vs. NLP_Pya_ hydrogen atoms (the radial distribution function accounts for the probability of finding a protein hydrogen atom at a given distance from the selected hydrogen atom of the inhibitor). As a result, the hydrogen atoms of the benzoic acid fragment were the closest to the protein residues (Fig 5B), in line with the STD-NMR experiments (Fig 4A). In addition, we also explored the possible binding modes of **6E11**, **6C3** and **7C8** to NLP_Pya_ central cavity. In this case we obtained a meta-stable binding pose exclusively for **7C8,** which remained bound for hundreds of ns between the NLP_Pya_ loops, before dissociating, while the two remaining compounds (**6E11**, **6C3**) dissociated from their initial docking poses within few ns of MD simulations (S5 Fig).

### Functional characterization of 6G7

**Fig 6:**
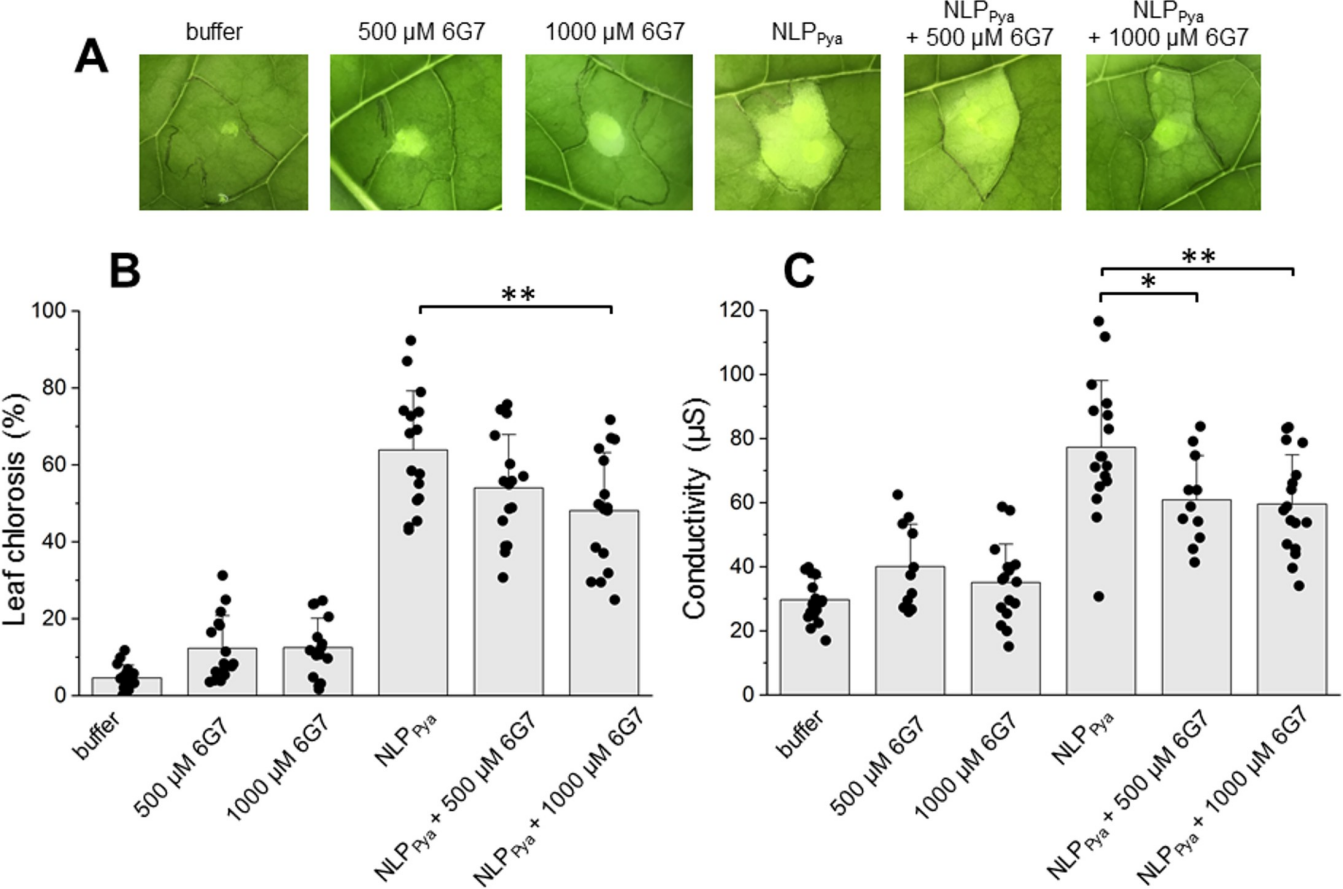
The inhibitory effect of the compound 6G7 on NLP toxicity. (A) The effects of 500 μM and 1 mM **6G7** on necrotic lesions formation induced by infiltrating tobacco leaves with 100 nM NLP_Pya_. **6G7** and protein were dissolved in 20 mM MES, 150 mM NaCl, pH 5.8, containing 10% DMSO (buffer). The upper part of the leaf was photographed after 24 h. The injected solution of protein caused leaf necrosis, whereas buffer or the compounds alone did not substantially affect the plant tissue. (B) Statistical analysis of leaf chlorosis developed 24 hours after infiltrating tobacco leaves with either buffer, **6G7** or 100 nM NLP_Pya_ in absence and presence of **6G7**. Values are means ± SD (n = 16), **P<0.01 vs. control (NLP_Pya_); Student’s t-test. Buffer, 20 mM MES, 150 mM NaCl, pH 5.8, 10% DMSO. (C) Quantification of cell death by means of ion leakage experiment measurement. Values are means ± SD (n = 12–18), *P<0.05, **P<0.01 vs. control (100 nM NLP_Pya_); Student’s t-test. Buffer, 20 mM MES, 150 mM NaCl, pH 5.8, 10% DMSO.

Finally, we performed thorough functional characterization of **6G7**. After 24 h, NLP_Pya_-induced leaf chlorosis was efficiently reduced in the presence of 1 mM **6G7**, dissolved in buffer containing 10% DMSO (Fig 6A and 6B). Cytolytic activity of NLPs can also be assayed by measuring ion leakage of tissue [20], where increase in electrolytic conductivity of water correlates with the amount of ion leakage from cells. The ability of **6G7** to reduce cytotoxic damage induced by NLP_Pya_ was assessed 2 h after incubation of treated leaf tissue in water. We confirmed that 500 μM and 1 mM **6G7** concentrations efficiently inhibit ion leakage induced by NLP_Pya_ from tobacco cells (Fig 6C).

## Discussion

There is a constant need for new agents to combat the most pressing plant pathogens. Thus, discovering novel inhibitors that target specific molecules or metabolic processes of pathogenic microorganisms is crucial. NLPs represent an important molecular target for phytopharmacological inhibition, as they are key virulence factors in plant pathogens and exhibit an extremely broad taxonomic distribution, occurring in both bacterial and eukaryotic microbes [5,24]. In this study, we propose a selection of small molecule compounds that could serve as inhibitors of NLP activity and might thus be used to control plant pathogens, particularly oomycetes of the genus *Phytophthora*.

Various compounds are used to control plant pathogens. Currently, pathogens such as *P*. *infestans*, which causes potato and tomato blight, are suppressed by organic (i.e., Mancozeb, Metalaxyl, or Fosetyl-Aluminum) or inorganic molecules (i.e., copper sulfate, copper hydroxide, or zinc ions). All these molecules act on proteins nonspecifically by chemically modifying certain amino acids. Mancozeb reacts with the thiol groups of cysteine residues, thereby inactivating proteins [28], and Metalaxyl inhibits RNA synthesis [29]. Fosetyl-Aluminum influences enzymatic activity [30] and was also reported to trigger host plant defense mechanisms [31]. Similarly, copper preparations have multilateral mode of action and act indiscriminately on all cells by impacting protein structure [32]. The use of such nonspecific compounds is problematic due to their nonspecific impact on plants, other organisms, and the environment in general.

Additionally, preventive fungicidal applications are frequently used due to the lack of effective methods for predicting plant pathogen outbreaks. For example, grapevine plantations are usually sprayed with fungicides up to four times before harvest to control the mold caused by *Botrytis cinerea* [33]. Numerous cases of resistance have been reported, including resistance to phenylamides (used to control *Phytophthora*) [34], resistance to azoles (used to control various pathogenic fungi and oomycetes) [35], and resistance to quinoline oxidation inhibitors [36]. Additionally, side effects of various fungicides have been reported. For example, Mancozeb is a broad-spectrum fungicide, which has been used for decades for diverse applications, including chemical control of the most important crops (i.e., potatoes, tomatoes, grapevines, and citrus fruits), yet has been reported to affect reproductive capacity in mammals [37].

In order to identify small molecular weight compounds that bind to NLPs, we utilized an SPR-based approach. SPR is often used for screening tests in the pharmaceutical and biotechnological industry, as it enables rapid initial testing of a large number of compounds as well as estimating the affinity and kinetic parameters of the interaction. We tested 587 unique and diverse compounds from our in-house library and identified three structurally distinct binders, **6G7**, **7C8**, and **6C3** (Figs 1 and 2), which were subsequently tested for NLP inhibition. All three compounds inhibited NLP_Pp_- and NLP_Pya_-induced necrosis (S1 and S6 Figs). To date, only one study has reported NLP inhibition. The compound dynasore reduced the necrosis-inducing action of BcNEP1, a NLP from *Botrytis cinerea*, but was ineffective against NLP_Pp_. However, dynasore likely inhibits the plant endocytotic pathway, and its direct interaction with NLP was not tested [38].

**7C8** exhibited the highest affinity for both tested NLPs with a K_D_ value of approximately 50 μM (Fig 2). However, **7C8** was only poorly soluble in the SPR running buffer containing 5% DMSO and only dissolved to a concentration of approximately 180 μM. The solubility of pharmacological compounds is a substantial problem, as more than 40% of new compounds are fairly insoluble in aqueous solutions [39]. Certain chemical modifications that could increase the solubility of **7C8** might also lower its high toxicity (S2 Fig). However, **7C8** did not significantly impact *Phytophthora* growth on potato leaves (Fig 3), whereas it proved to be efficient in inhibiting necrosis (S1 Fig). The SPR response of **6C3** binding to NLP was 100 times higher than the expected maximal response (Fig 2), indicating promiscuous binding of this compound. Such molecules typically aggregate into clusters of up to 100 nm in size, bind nonspecifically to the protein, and inhibit its activity. A number of small compounds are prone to aggregation and were thus not further considered [40]. **6G7** exhibits the most appropriate properties, as it exhibited affinity in the micromolar range for two NLPs (Figs 2 and 4), inhibited necrosis (S1 and S6), significantly inhibited the growth of the NLP-producing oomycete pathogen *P*. *infestans* (Fig 3), and was non-toxic to Caco-2 cells (S2 Fig). In addition, this was the only compound which in all-atoms MD simulations remained stably bound to the NLP_Pya_ cavity implicated in GIPC recognition (Fig 5). Taking into consideration all of the above as well as the structural simplicity of this anthranilic acid derivative, which enables further investigation of its structure-activity relationship, **6G7** represents the most promising candidate molecule for developing novel phytopharmaceutical substances that could inhibit NLP-producing pathogens.

Inhibiting the activity of cytolytic proteins is an important strategy to prevent the undesirable effects of toxic molecules in any physiological setup [41]. In this work, we propose targeting effector molecules produced by different microbial pathogens in the development of specific phytopharmaceutical compounds. Recent structural investigations on the interaction of NLPs with plant cell receptors [21] may aid in the structure-based design of new compounds. As microbes secrete NLPs into the apoplast of their host plants, phytopharmaceutical compounds targeting this major virulence factor may efficiently safeguard plant health. Our study provides chemical lead structures for plant pest control. Chemical derivatization is now required to optimize the biosafety, biodegradability, bioavailability, solubility, and efficacy of these phytoprotective substances.

## Materials and methods

### Heterologous expression and purification of NLPs

NLP from the oomycete *Pythium aphanidermatum* (NLP_Pya_) was prepared using the heterologous expression system for protein production in *Escherichia coli* [20]. NLP from the oomycete *Phytophthora parasitica* (NLP_Pp_) was prepared in the yeast *Pichia pastoris* as described previously [12]. Proteins were stored at –20°C.

### Characterization and synthesis of compounds

Compounds **6G7** (2-((4-methoxyphenyl)amino)benzoic acid) and **6E11** (2-methyl-5-((1*S*,2*R*,3*R*)-1,2,3,4-tetrahydroxybutyl)furan-3-carboxylic acid) were purchased from Enamine, and **6C3** ((4*R*)-2-(6-methyl-4-oxo-4*H*-chromen-3-yl)thiazolidine-4-carboxylic acid) was purchased from Vitas-M Laboratory. **7C8** (2-(2-(1*H*-indol-3-yl)acetamido)phenethyl 2-(1*H*-indol-3-yl)acetate) was synthesized at the Faculty of Pharmacy, University of Ljubljana, as described below. The reaction was monitored using analytical thin-layer chromatography (TLC) plates (Merck 60 F254, 0.20 mm), and the components were visualized under UV light and/or by ninhydrin staining. Flash column chromatography was performed on Merck Silica Gel 60 (particle size 0.040−0.063 mm; Merck, Germany). Prior to use in the biochemical assays, all compounds were characterized spectroscopically (S2 Methods), and their purity was determined by high-performance liquid chromatography (HPLC). ^1^H and ^13^C nuclear magnetic resonance (NMR) spectra were recorded on a Bruker Avance III 400 MHz spectrometer at 295 K. The chemical shifts (δ) were reported in parts per million (ppm) and were referenced to the deuterated solvent used. The coupling constants (*J*) were provided in Hz, and the splitting patterns were designated as follows: s, singlet; br s, broad singlet; d, doublet; app d, apparent doublet; dd, double doublet; ddd, doublet of doublet of doublets; t, triplet; m, multiplet. Infrared spectra were obtained on a Thermo Nicolet Fourier transform infrared spectrometer using the attenuated total reflection (ATR) technique. Mass spectra data and high-resolution mass measurements were performed on a VG-Analytical Autospec Q mass spectrometer at the Jožef Stefan Institute, Ljubljana, Slovenia. Analytical reversed-phase HPLC for the test compounds was performed on an Agilent 1100 LC modular system that was equipped with a photodiode array detector set at 254 nm. An Agilent Eclipse Plus C18 column (150 × 4.6 mm; 5 μm) was used, which was thermostated at 25°C, with a flow rate of 1.0 mL/min and a sample injection volume of 10 μL. An eluent system of (A) H_2_O with 0.1% TFA or (B) MeCN was used. The following gradient was applied for compounds **6G7** and **7C8**: 0−3 min, 40% B; 3−16 min, 40% B ➔ 90% B; 16−19 min, 90% B; 19−20 min, 90% B ➔ 40% B; run time, 20 min. The following gradient was applied for compound **6C3**: 0−3 min, 10% B; 3−12 min, 10% B ➔ 90% B; 12−14 min, 90% B; 14−15 min, 90% B ➔ 10% B; run time, 15 min. The following gradient was applied for compound **6E11**: 0−5 min, 5% B; 5−10 min, 5% B ➔ 90% B; 10−11 min, 90% B; 11−12 min, 90% B ➔ 5% B; run time, 12 min. The purities of the test compounds used for the biological evaluations were >95%, as determined by HPLC.

### The preparation of compound 7C8

Solutions of 1-ethyl-3-(3-dimethylaminopropyl)carbodiimide (537 mg, 2.94 mmol) and Et_3_N (938 μL, 683 mg, 6.78 mmol) were added to compound **1** (396 mg, 2.26 mmol) in dry *N*,*N*-dimethylformamide (10 mL) at 0°C, and the resulting mixture was stirred at 0°C for 10 min. Then compound **2** (341 mg, 2.49 mmol) was slowly added, followed by the addition of hydroxybenzotriazole (415 mg, 2.71 mmol). After initial stirring at 0°C for 30 min, the reaction mixture was left to stir at room temperature for 48 h. After the reaction was complete (monitored by TLC), a saturated aqueous solution of citric acid (30 mL) was added. The mixture was transferred into a separation funnel and extracted with EtOAc (2 × 50 mL). The combined organic phases were washed with a saturated aqueous solution of NaHCO_3_ (2 × 50 mL) and brine (1 × 50 mL), dried with Na_2_SO_4_, filtered, and evaporated under reduced pressure. The crude product was purified by column chromatography (EtOAc/*n*-hexane, 1/1) to yield 295 mg (29%) of pure **7C8** (S1 Scheme).

### Surface plasmon resonance

Biacore T100 equipped with the Series S Sensor chip CM5 (GE Healthcare) was used to assess the binding of compounds to NLP_Pya_ and NLP_Pp_. The proteins were covalently immobilized to the chip using amine coupling. The surface was activated with a 10 min injection of a mixture of 1-ethyl-3-(3-dimethylaminopropyl) carbodiimide hydrochloride / *N*-hydroxysuccinimide (1:1). NLPs were immobilized on the second flow cell. The first flow cell was left empty to control any nonspecific compound binding to the dextran matrix. Both cells were finally blocked with a 7 min injection of ethanolamine. In total, 587 in-house compounds (Faculty of Pharmacy, University of Ljubljana) were dissolved in DMSO as stock solutions at a concentration of 10 mM and diluted in 50 mM 2-(N-morpholino)ethanesulfonic acid (MES) and 150 mM NaCl to a final concentration of 5% DMSO (pH 5.8) prior to SPR experiments. After washing the sensor surface with running buffer (50 mM MES, 150 mM NaCl, 5% DMSO, pH 5.8), the compounds were tested at two different concentrations: 20 and 200 μM. Certain compounds were tested at lower concentrations owing to their lower solubility in the running buffer. Each compound was injected for 1 min at a flow rate of 30 μL/min, and dissociation was monitored for another minute. The compounds that were not completely soluble in the running buffer exhibited saw-tooth like curves and were omitted from further analysis. In the next set of experiments, 67 selected compounds were titrated. The samples were injected for 1 min at a flow rate of 30 μL/min, and dissociation was monitored for another minute. The samples were typically injected at the following concentrations: 0, 12.5, 25, 50, 100, and 200 μM. At the end of each concentration series, 25 μM of compound was injected again to control the activity of the surface. Regeneration between individual injections was not needed for molecules that completely dissociated from the protein. When complete dissociation was not achieved, short pulses of 0.1% SDS were included. The obtained sensorgrams were analyzed using the Biacore T100 Evaluation software, and the steady-state affinity binding model was used to calculate affinity constants.

### Infiltration assay

Tobacco plants (*Nicotiana tabacum* ‘White Burley’) were grown in a controlled growth chamber with supplementary light (70−90 μMm^-2^s^-1^) and a 16 h photoperiod with 22°C day and 20°C night temperatures at 75±2% humidity. Using blunt-ended syringe pressure infiltration, 100 μL of solution (either 400 nM NLP_Pp_ or 250 nM NLP_Pya_ in ultrapure water in the absence or presence of the compounds) was infiltrated abaxially into the leaves of 5−7-week-old tobacco plants (*Nicotiana tabacum* ‘White Burley’). 5% DMSO, NLP solutions, and appropriate compound dilutions were used as controls. The area on the upper side of the leaf, which was infiltrated with solutions, was labeled and checked for the presence of necrosis after 24 h. To thoroughly assess the effect of compound **6G7** on the level of leaf necrosis/chlorosis, 50 μL of 100 nM NLP_Pya_ solution without/with 500 μM or 1 mM **6G7** in 20 mM MES, 150 mM NaCl, pH 5.8, containing 10% DMSO was infiltrated. The level of leaf chlorosis was estimated using ImageJ software by calculating the ratio of the chlorotic area to the whole infiltrated area, both measured in squared pixels. Each value represents the average of sixteen infiltrations.

### *P*. *infestans* growth on potato leaves

Leaves were removed from potato plants (*Solanum tuberosum* var. Desirée) and infected with a 10 μL drop that contained 5 × 10^4^ mL^-1^ of zoospores of the pathogen *P*. *infestans* (strain 88069). Drops also contained 1 mM solutions of **6E11** (control), **6C3**, **6G7** or **7C8**. The infected leaves were incubated for 4 days in a humid atmosphere at 18°C, after which they were photographed. The infected areas of the leaves were removed (as discs of 12 mm in diameter) for DNA extraction by the established procedure [42]. DNA quantification was performed with real-time PCR (iQ5 iCycler, Bio-Rad) using 1 μL of DNA in 20 μL of buffer that contained SYBR green dye (Thermo Scientific). The oligonucleotides Pi08-3-3-fwd (5’-CAATTCGCCACCTTCTTCGA-3’) and Pi08-3-3-rev (5’-GCCTTCCTGCCCTCAAGAAC-3’) for amplification were selected based on the repetitive sequences in the genome of *P*. *infestans* [14]. The amount of DNA was determined according to the calibration curve in the range of 0.001−100 ng of DNA obtained from the mycelium of *P*. *infestans* (spectrophotometric determination of DNA concentration, NanoDrop 2000, Thermo Scientific). The following conditions were used for amplification: 10 min at 95°C, 40 cycles of 10 s at 95°C, 15 s at 59°C, and 20 s at 72°C. The means of three technical repeats were determined.

### NMR assignment for saturation transfer difference (STD)-NMR experiments

The 1D and 2D NMR spectra of **6G7** were acquired on an Agilent Technologies DD2 600 MHz NMR spectrometer at 25°C using a 5 mm ^1^H (^13^C/^15^N) ^13^C-enhanced Cold Probe. Data acquisition and processing was performed with software VNMRJ version 3.2 and MestReNova version 10.0.2–15465. The spectra for assignment were recorded in ^2^H_2_O, 25 mM Tris-*d*_11_, 150 mM NaCl, pH 7.5, 5% DMSO-*d*_6_ at 25°C. Chemical shifts were referenced to the residual solvent signal of DMSO-*d*_6_ at δ 2.5 ppm for ^1^H (600 MHz) and δ 39.5 ppm for ^13^C (150 MHz). The ^1^H and ^13^C resonances of **6G7** have been assigned based on the analysis of 1D ^1^H and ^13^C spectra and ^13^C-^1^H correlations in 2D HSQC and HMBC spectra. The atom numbering used in the NMR assignment is indicated in Fig 4A.

STD-NMR experiments were performed with 8 μM NLP_Pya_. A pseudo-2D version of a STD-NMR pulse sequence with DPFGSE water suppression for the interleave acquisition of on- (δ 0.2 ppm) and off-resonance (δ 40 ppm) spectra was used at 25°C with 512 scans. A 30 ms spin-lock filter was used for protein signal suppression. The STD spectra were obtained by subtracting the saturated spectra from the reference spectra. The STD effect was calculated by (I_0_-I_sat_)/I_0_, where I_0_ is the signal intensity in the off-resonance spectrum, I_sat_ is the signal intensity in the on-resonance spectrum, and I_0_-I_sat_ is the intensity of the STD-NMR spectrum. Appropriate control experiments in the absence of protein were performed to assure the absence of direct irradiation of the ligand. In the titration study, five STD experiments were performed with varying ligand concentrations (40, 80, 240, 400, and 560 μM).

The STD amplification factor (STD-AF) was determined according to Eq 1, where [L] is the ligand concentration, and [P] is the NLP_Pya_ concentration.

$$STD-AF=\frac{{I_{0}-I}_{sat}}{I_{0}}*\frac{\left[L\right]}{\left[P\right]}$$ (1)

The hyperbolic behavior of the curves obtained from plotting STD-AF as a function of ligand concentration is appropriately described by Eq 2, where STD-AF is the STD amplification factor, α_STD_ is the maximum amplification factor, [L] is the ligand concentration, and K_D_ is the dissociation constant.

$$STD-AF=\frac{\alpha_{STD}*[L]}{K_{D}+[L]}$$ (2)

Non-linear least-squares curve-fitting to Eq 2 was performed with Origin 8.1 software.

### Binding site mapping and molecular docking

The FTMap program [25] was used to identify the potential ligands’ binding sites with small molecular probes (acetaldehyde, acetamide, acetone, acetonitrile, benzaldehyde, benzene, cyclohexane, dimethyl ether, ethane, isobutanol, isopropanol, methylamine, N,N- dimethylformamide, phenol and urea) on the X-ray structure of NLP_Pya_ (PDB ID 3GNZ) and on two representative structures selected from a cluster analysis of a μs-long MD simulations of NLP_Pya_ in explicit solvent obtained in our previous study [13]. Three potential binding sites were identified: a central cavity (Site 1) comprising D93, D104, Y105, E106, N107, H128, S126, and N194, which coincides with the cavity implicated in GIPC binding [21], an upper cavity, named Site 2, comprising T20, G76 and Y82, and an upper-right cavity, named Site 3, comprising I120, L142 and I145 residues of NLP_Pya_ (S3 Fig). The binding mode of four compounds was investigated: **6G7**, **6E11**, **6C3** and **7C8.** They were initially pre-processed by Schrodinger Suite 2017–1 Epik tool [43] to establish their most likely protonation state at physiological pH. Next, the compounds were docked into to the NLP_Pya_ crystal structure, with the Glide program, considering 20 possible conformations of each ligand [26]. All crystallographic waters were removed and the docking grid boxes were set to a radius of 15 Å. The resulting molecules were sorted by GlideScore scoring function and the top-ranked binding poses were further refined by performing MD simulations.

### System preparation for simulations

The inhibitor/NLP_Pya_ complex structures were built on the crystal structure of NLP_Pya_ (PDB ID 3GNZ), where the protonation states of ionizable residues were assigned on the basis of the Propka program [44] and all the crystal waters were retained. The topologies were built with Amber ff14SB force field for proteins [45] using the Ambertools 18 module of AMBER program [46]. The system was solvated by adding a layer of 12 Å of TIP3P water molecules in each direction [47], leading to a total of 36961 atoms for NLP_Pya_. Mg^2+^ ion was described with Allner parameters [48] and Cl^-^ ions were added to achieve charge neutrality using the ion parameters of ref. [49]. The partial ESP charges of all ligands were obtained by performing population analysis according to the Merz-Kollman scheme on their optimized geometry at Hartree-Fock level of theory, using 6–31 G* basis set with the Gaussian09 program [50]. Next, RESP charges were generated with the Antechamber module of Amber18 and the other ligand’s force field parameters were obtained with Antechamber module, on the previously optimized geometries.

### Molecular dynamics simulations

After the initial minimization, each ligand/NLP_Pya_ system was heated up to 300K over 10 ns, while imposing positional restraints of 250 kcal/molÅ^2^ on the heavy atoms. Subsequently, restraints were slowly removed and a productive MD simulation was run on the isothermal-isobaric ensemble (NPT) using periodic boundary condition. The temperature control (300K) was performed by Langevin thermostat [51] with a collision frequency of 1 ps^-1^, and pressure control (1 atm) was accomplished by Berendsen barostat [52]. The SHAKE algorithm [53] was used to constrain the bonds involving hydrogen atoms and the particle mesh Ewald method [54] to account for long-range electrostatic interactions with a cutoff of 10 Å. An integration time step of 2 fs was used. The Radial distribution function was calculated in the cpptraj module of Ambertools 18 [46]. Only **6G7** resulted to be stable in a 1 μs-long trajectory.

### QM/MM molecular dynamics simulations

After the classical MD simulations the **6G7**/NLP_Pya_ adduct was relaxed by 5 ps of QM (Born–Oppenheimer)/MM MD simulations performed with the CP2K 6.1 program [55] to properly describe the metal-ligand interactions [56]. The QM region comprised the sidechain atoms of Asp93 and Asp104 residues of NLP_Pya_, the **6G7** compound, the Mg^2+^ ion and three water molecules (51 atoms), while the rest of the system (the protein and the explicit solvent) was treated at MM level with the same force field of the classical MD simulations. The QM region was simulated in a cubic box with sides of 21 Å and described at the DFT-BLYP level by employing a dual Gaussian-type/plane waves basis set (GPW) [57]. We employed a double zeta (MOLOPT) basis set [58], along with an auxiliary PW basis set with a density cutoff of 320 Ry and Goedecker–Teter–Hutter (GTH) pseudopotentials [59]. The valences of terminal QM atoms were saturated by using capping hydrogen atoms. All QM/MM MD simulations were performed using an integration time step of 0.5 fs in the NVT ensemble. Constant temperature was maintained by employing a Nosé–Hoover thermostat [60].

### Ion leakage

Tobacco leaves were infiltrated with 100 nM NLP_Pya_ in 20 mM MES, 150 mM NaCl pH 5.8, preincubated for 10 min with 500 μM or 1 mM **6G7**. Final concentration of DMSO in solution was 10%. After 10 min incubation, 2 leaf disks were punched out (Ø 6 mm) and transferred to 2 mL of deionized distilled water. After 30 min of shaking at 430 rpm, leaf disks were transferred to 1 mL of fresh deionized distilled water. Conductivity was measured after 2 h using SevenCompact Cond meter (Mettler Toledo). Mock treated samples were discs from leaves infiltrated with buffer containing 10% DMSO or **6G7**. Each value represents the average of 12–18 independent experiments.

## Supporting information

S1 Fig Infiltration experiments. (A) The control experiments of infiltrating tobacco leaves with either 5% DMSO, 400 nM NLP_Pp_, 200 nM NLP_Pya_, or 1 mM **6G7**, **6C3**, **7C8**, or **6E11** in 5% DMSO. The upper part of the leaf was photographed after 24 h. The injected solutions of both NLPs caused leaf necrosis, whereas 5% DMSO or the compounds alone did not affect the plant tissue. (B) NLP_Pp_-induced necrosis was inhibited by different concentrations of **6G7**, **6C3**, and **7C8**. **6E11** was not affective against NLP toxicity. (C) NLP_Pya_-induced necrosis was inhibited by 1 mM **6G7** and **6C3** and 200 μM **7C8**.
(TIF)

S2 Fig The effects of 7C8, 6C3, and 6G7 on the viability of Caco-2 cells. The compounds were tested at the concentrations indicated. The treatments with only DMSO used the concentration of DMSO necessary to solubilize each compound at its highest concentration. The DMSO concentration in the treatments was reduced proportionally with the reduced compound concentrations.
(TIF)

S3 Fig Binding sites mapping. Potential inhibitors’ binding sites (Sites 1, 2 and 3) mapped with various small molecule probes (acetaldehyde, acetamide, acetone, acetonitrile, benzaldehyde, benzene, cyclohexane, dimethyl ether, ethane, isobutanol, isopropanol, methylamine, N,N- dimethylformamide, phenol and urea) depicted with coloured ball and sticks on the NLP_Pya_ crystal structure (PDB ID 3GNZ) and two of the most populated clusters from molecular dynamics simulation trajectory.
(TIF)

S4 Fig Binding sites assessment. (A) Docking possess of the **6G7** compound in the central Mg^2+^ containing binding cavity, Site 1 (left panel), in Site 2 (middle panel) and in Site 3 (right panel) with docking scores of −4.79 kcal/mol, - 2.53 kcal/mol and −2.45 kcal/mol, respectively. (B) Binding modes of **6G7** in Sites 1, 2 and 3 as obtained after the Molecular Dynamic (MD) simulations. Only the binding pose in the central cavity Site 1 remained stable during a μs-long MD simulation, whereas the other two poses dissociated within the few ns of MD run.
(TIF)

S5 Fig Binding mode of 6E11, 6C3 and 7C8 compounds. Docking poses of the **6E11** (A), **6C3** (B) and **7C8** (C) compounds. During Molecular Dynamics simulations only the **7C8** ligand obtained a meta-stable binding pose between the loops of NLP_Pya_ (D), whereas **6E11** and **6C3** quickly dissociated from the initial binding sites.
(TIF)

S1 Methods Assessment of compound toxicity. (DOCX)

S2 Methods NMR characterization of the compounds. (DOCX)

S1 Scheme Synthesis of 7C8. Reagents and conditions: 1-ethyl-3-(3-dimethylaminopropyl)carbodiimide, hydroxybenzotriazole, Et_3_N, *N*,*N*-dimethylformamide, 0°C to room temperature, 24 h.
(TIF)
