# Correction: Nep1-like proteins as a target for plant pathogen control

**DOI:** 10.1371/journal.ppat.1013758

**Published:** 2025-12-17

**Authors:** The PLOS Pathogens Editors

After publication of this article [1], the corresponding author provided the underlying data supporting the published results. With the provision of these data, available in S1-S2 Files with this notice, this article [1] complies with PLOS’ Data Availability Policy.

The corresponding author clarified that the data in Fig 2 was plotted such that the surface plasmon resonance signal in the association phase (times between 0–60 s) was optimally represented in the plots, meaning some of the spikes are not shown in Fig 2. An updated caption for Fig 2 is provided here.

For Fig 3, the corresponding author stated that 10 replicates are present in panel A of Fig 3, and 9 or 8 replicates per inhibitor are present in panel B (two for the control with no inhibitor) as indicated in S1 File.

The corresponding author does not agree that a Correction is warranted to address the Data Availability issue.

**Fig 2:**
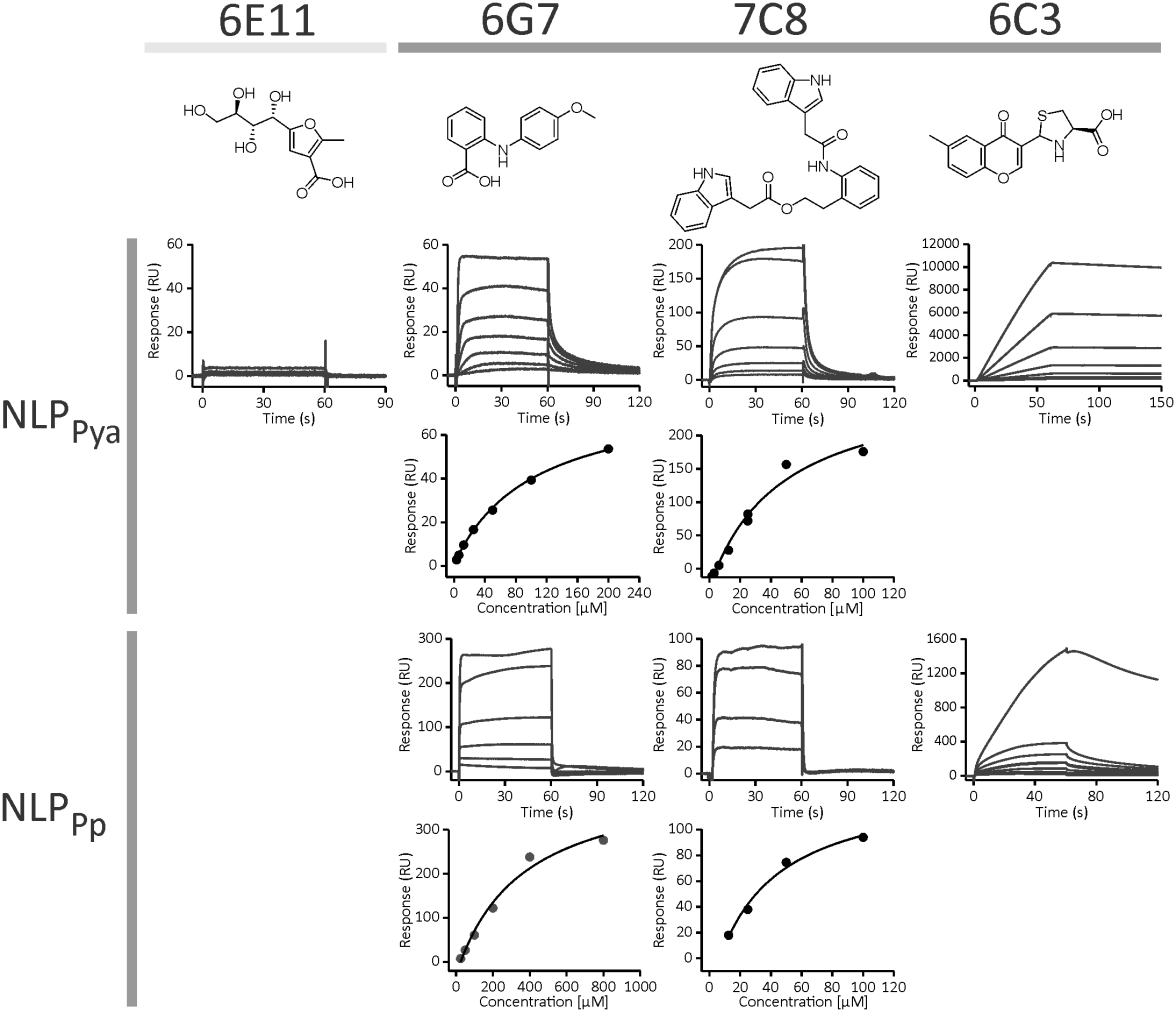
Binding of compounds 6G7, 7C8, and 6C3 to NLP_Pya_ and NLP_Pp_. The most promising binders were compounds 6G7 and 7C8. The binding response of **6C3** exceeded the expected response for a 1:1 interaction, indicating multi-site binding. Compound **6E11** was used as a control. Data were fit to the steady-state affinity model. Fig 2 shows graphs which, for reasons of clarity, do not show the entire range of data. Please see S1 File for details.

## Supporting information

S1 File Underlying dataset supporting the results in Figures S2, 1A-C, 2, 3B, 4A-C, 5B and 6B-C, including values and calculations for mean, standard deviation and t-test. (XLSX)

S2 File Underlying data supporting NMR spectra shown in Figures 4A and B. (ZIP)
