# Supplementary figures and images for: Nep1-like proteins as a target for plant pathogen control
Source: PLoS Pathog. 2021 Apr 15;17(4):e1009477. doi: 10.1371/journal.ppat.1009477 (PMC8078777; doi:10.1371/journal.ppat.1009477)

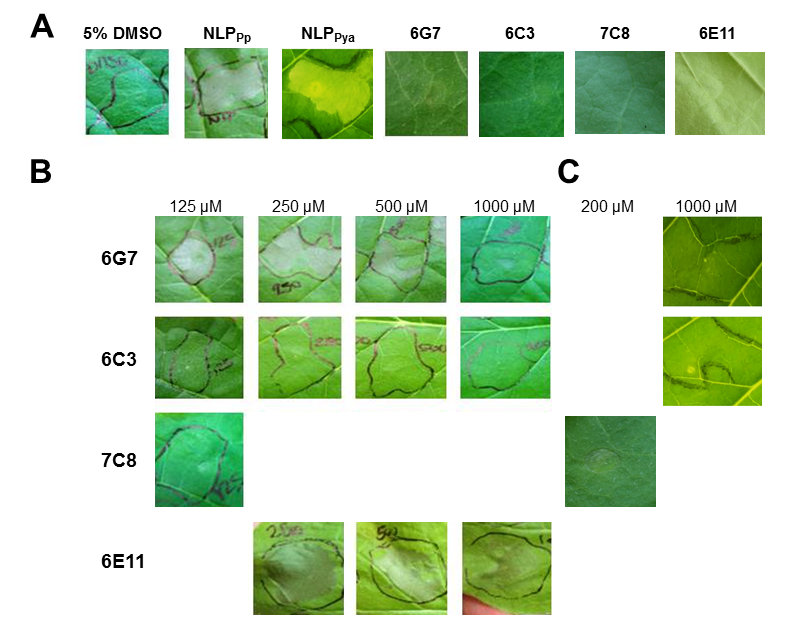

Supplement: S1 Fig — (A) The control experiments of infiltrating tobacco leaves with either 5% DMSO, 400 nM NLPPp, 200 nM NLPPya, or 1 mM 6G7, 6C3, 7C8, or 6E11 in 5% DMSO. The upper part of the leaf was photographed after 24 h. The injected solutions of both NLPs caused leaf necrosis, whereas 5% DMSO or the compounds alone did not affect the plant tissue. (B) NLPPp-induced necrosis was inhibited by different concentrations of 6G7, 6C3, and 7C8. 6E11 was not affective against NLP toxicity. (C) NLPPya-induced necrosis was inhibited by 1 mM 6G7 and 6C3 and 200 μM 7C8. (TIF) [file ppat.1009477.s001.tif]

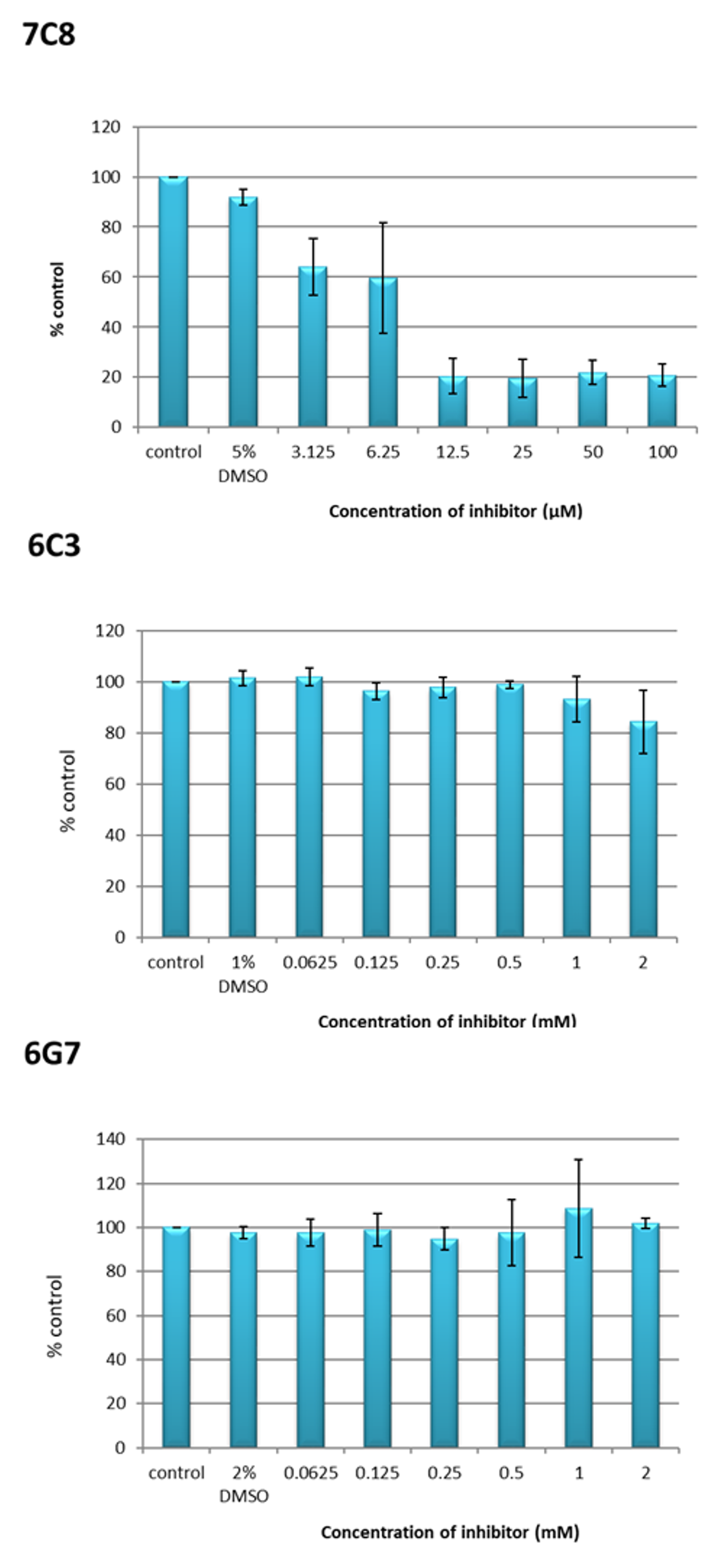

Supplement: S2 Fig — The compounds were tested at the concentrations indicated. The treatments with only DMSO used the concentration of DMSO necessary to solubilize each compound at its highest concentration. The DMSO concentration in the treatments was reduced proportionally with the reduced compound concentrations. (TIF) [file ppat.1009477.s002.tif]

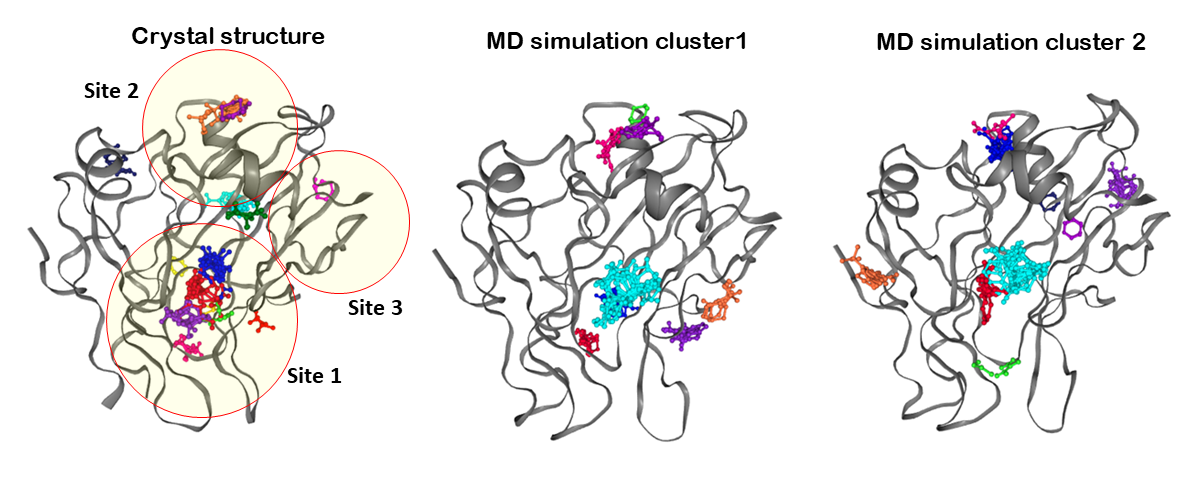

Supplement: S3 Fig — Potential inhibitors’ binding sites (Sites 1, 2 and 3) mapped with various small molecule probes (acetaldehyde, acetamide, acetone, acetonitrile, benzaldehyde, benzene, cyclohexane, dimethyl ether, ethane, isobutanol, isopropanol, methylamine, N,N- dimethylformamide, phenol and urea) depicted with coloured ball and sticks on the NLPPya crystal structure (PDB ID 3GNZ) and two of the most populated clusters from molecular dynamics simulation trajectory. (TIF) [file ppat.1009477.s003.tif]

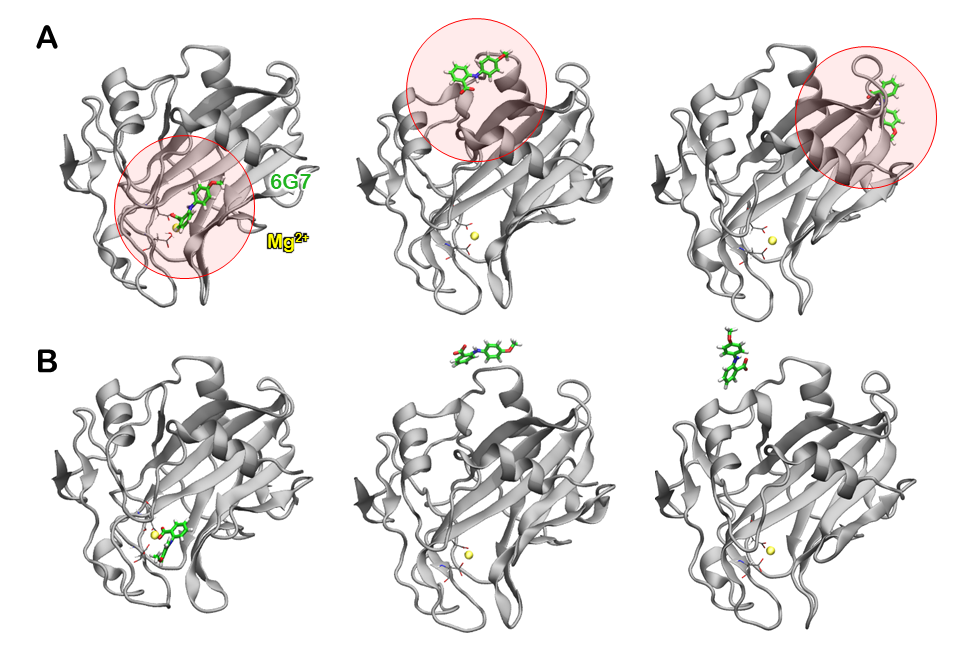

Supplement: S4 Fig — (A) Docking possess of the 6G7 compound in the central Mg2+ containing binding cavity, Site 1 (left panel), in Site 2 (middle panel) and in Site 3 (right panel) with docking scores of −4.79 kcal/mol, - 2.53 kcal/mol and −2.45 kcal/mol, respectively. (B) Binding modes of 6G7 in Sites 1, 2 and 3 as obtained after the Molecular Dynamic (MD) simulations. Only the binding pose in the central cavity Site 1 remained stable during a μs-long MD simulation, whereas the other two poses dissociated within the few ns of MD run. (TIF) [file ppat.1009477.s004.tif]

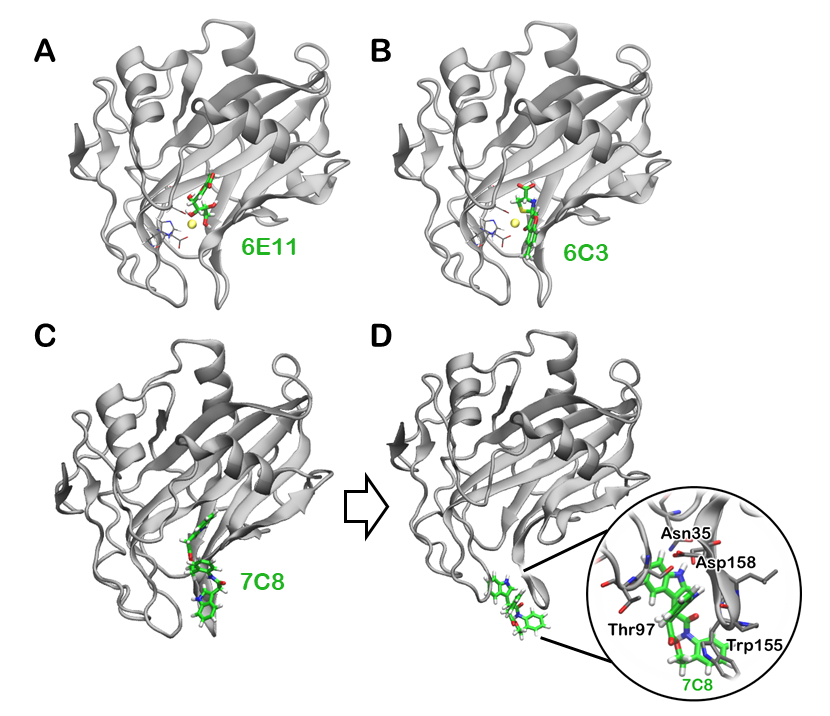

Supplement: S5 Fig — Docking poses of the 6E11 (A), 6C3 (B) and 7C8 (C) compounds. During Molecular Dynamics simulations only the 7C8 ligand obtained a meta-stable binding pose between the loops of NLPPya (D), whereas 6E11 and 6C3 quickly dissociated from the initial binding sites. (TIF) [file ppat.1009477.s005.tif]

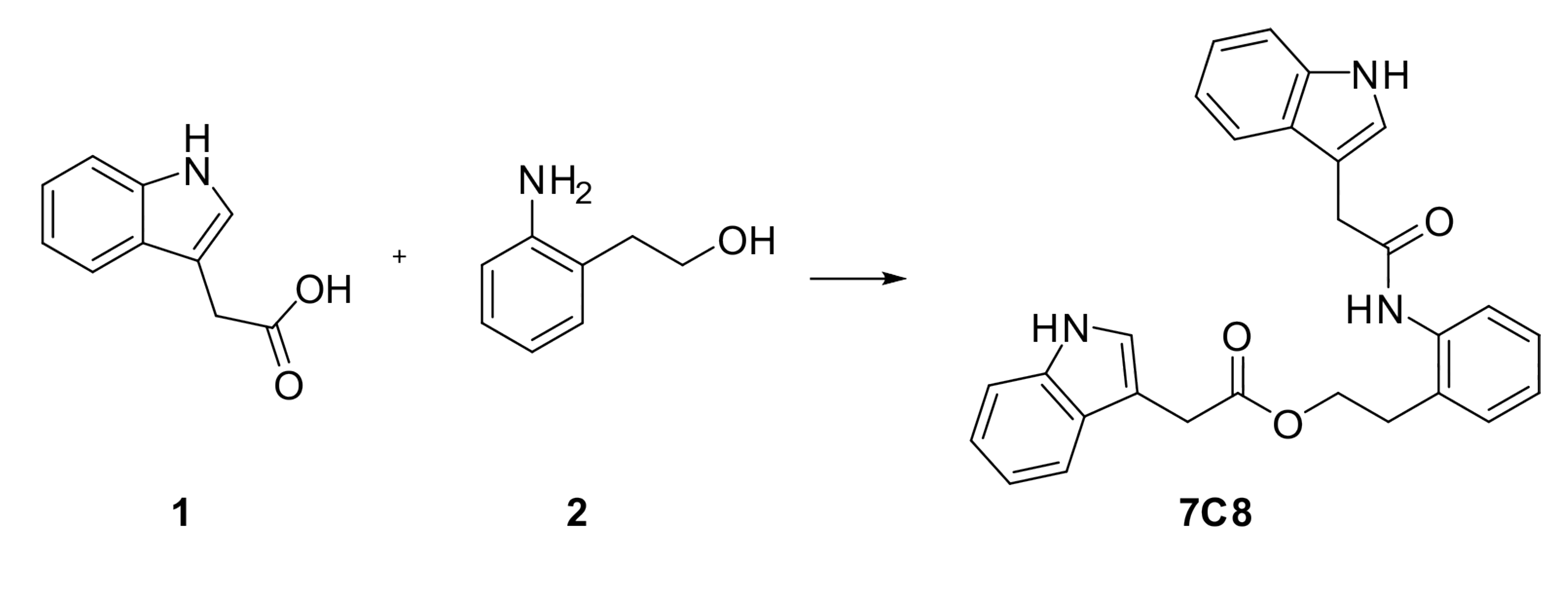

Supplement: S1 Scheme — Reagents and conditions: 1-ethyl-3-(3-dimethylaminopropyl)carbodiimide, hydroxybenzotriazole, Et3N, N,N-dimethylformamide, 0°C to room temperature, 24 h. (TIF) [file ppat.1009477.s008.tif]
