# Supplementary material for: Nep1-like proteins as a target for plant pathogen control
Source: PLoS Pathog. 2021 Apr 15;17(4):e1009477. doi: 10.1371/journal.ppat.1009477 (PMC8078777; doi:10.1371/journal.ppat.1009477)
Supplement: S1 Methods — (DOCX) [file ppat.1009477.s006.docx]

## S1 Methods

## Assessment of compound toxicity

Caco-2 cells were seeded onto microtiter 96-well plates at a density of 70,000 cells/mL in 50 µL of minimal essential medium (Sigma) supplemented with 10% fetal bovine serum (Sigma), 1% L-glutamine (Gibco), and 1% non-essential amino acids (Sigma). Cells were incubated for two days at 37 °C in a humidified atmosphere containing 5% CO_2_. Then the cells were treated with the selected compounds at different concentrations: **6G7** and **6C3** were added at concentrations of 0.0625, 0.125, 0.25, 0.5, 1, and 2 mM, and **7C8** at concentrations of 3.125, 6.25, 12.5, 25, 50, and 100 μM. The final DMSO concentration in the medium was adjusted as indicated by the solubility of individual compounds and was reduced proportionally with serial dilutions. The highest DMSO concentration in assays was 5%. After overnight incubation, the cell viability was assessed by the MTT assay. Briefly, 3-(4,5-dimethylthiazol-2-yl)-2,5-diphenyltetrazolium bromide (MTT) reagent was added to each well at a final concentration of 1 mg/mL and incubated for 3 h. The medium was then removed, and the formazan product of living cells was dissolved in DMSO. The absorbance was measured at 570 nm with a Fluostar Galaxy microplate reader. Three independent experiments with the compounds **6C3** and **7C8** and four independent experiments with the compound **6G7** were performed.
