# Supplementary material for: Nep1-like proteins as a target for plant pathogen control
Source: PLoS Pathog. 2021 Apr 15;17(4):e1009477. doi: 10.1371/journal.ppat.1009477 (PMC8078777; doi:10.1371/journal.ppat.1009477)
Supplement: S2 Methods — (DOCX) [file ppat.1009477.s007.docx]

## S2 Methods

## NMR characterization of the compounds

### NMR characterization of (4*R*)-2-(6-methyl-4-oxo-4*H*-chromen-3-yl)thiazolidine-4-carboxylic acid (6C3)

White solid, a mixture of two diastereoisomers with a 1.8:1 ratio. ^1^H NMR (400 MHz, DMSO-*d_6_*) for the major diastereoisomer δ (ppm) = 2.42 (s, 3H, Ar-CH_3_), 2.87 (dd, part A of AMX system, *J*_A,M_ = 10.2 Hz, *J*_A,X_ = 6.8 Hz, 1H, SCH_2_CH), 3.17 (dd, part M of AMX system, *J*_M,A_ = 10.2 Hz, *J*_M,X_ = 6.8 Hz, 1H, SCH_2_CH), 4.13 (dd, part X of AMX system, *J*_X,A_ = 6.8 Hz, *J*_X,M_ = 6.8 Hz, 1H, SCH_2_CH), 5.71 (d, *J* = 1.0 Hz, 1H, SCHNH), 7.54-7.62 (m, 2H, Ar-H), 7.82-7.85 (m, 1H, Ar-H), 8.27 (d, *J* = 1.0 Hz, 1H, Ar-H), resonances for NH and COOH missing; ^1^H NMR (400 MHz, DMSO-*d_6_*) for the minor diastereoisomer δ (ppm) = 2.43 (s, 3H, Ar-CH_3_), 2.91 (dd, part A of AMX system, *J*_A,M_ = 10.0 Hz, *J*_A,X_ = 9.3 Hz, 1H, SCH_2_CH), 3.31 (dd, part M of AMX system, *J*_M,A_ = 10.0 Hz, *J*_M,X_ = 6.4 Hz, 1H, SCH_2_CH, overlapping with water in DMSO), 3.85 (dd, part X of AMX system, *J*_X,A_ = 9.3 Hz, *J*_X,M_ = 6.4 Hz, 1H, SCH_2_CH), 5.49 (d, *J* = 0.5 Hz, 1H, SCHNH), 7.61-7.68 (m, 2H, Ar-H), 7.85-7.87 (m, 1H, Ar-H), 8.57 (d, *J* = 0.6 Hz, 1H, Ar-H), resonances for NH and COOH missing; ^13^C NMR (100 MHz, DMSO-*d_6_*) for the major diastereoisomer δ (ppm) = 20.41, 36.85, 63.22, 64.53, 118.20, 122.66, 123.99, 124.74, 134.96, 135.32, 152.26, 154.05, 172.59, 175.76; ^13^C NMR (100 MHz, DMSO-*d_6_*) for the minor diastereoisomer δ (ppm) = 20.44, 37.74, 64.94, 65.70, 118.24, 121.08, 122.83, 124.10, 135.26, 135.52, 153.86, 154.64, 172.32, 175.76; IR (ATR) υ = 2361, 1712, 1638, 1614, 1590, 1484, 1433, 1375, 1316, 1295, 1231, 1175, 828, 804, 791, 771 cm^–1^; HRMS (ESI) m/z calculated for C_14_H_14_NO_4_S [M+H]^+^ 292.0644, found 292.0642; purity by HPLC: 96.95% (peaks of diastereoisomers were overlapping).

### NMR characterization of 2-Methyl-5-((1*S*,2*R*,3*R*)-1,2,3,4-tetrahydroxybutyl)furan-3-carboxylic acid (6E11)

Off-white solid. ^1^H NMR (400 MHz, DMSO-*d_6_*) δ (ppm) = 2.48 (s, 2H, Ar-CH_3_), 3.40 (dd, *J*_1_ = 10.8 Hz, *J*_2_ = 5.3 Hz, 1H, H of CH_2_OH, overlapping with water in DMSO), 3.44-3.52 (m, 2H, furan-CH(OH)CH(OH)CH(OH) and H of CH_2_OH), 3.55-3.60 (m, 1H, furan-CH(OH)CH(OH)CH(OH)), 4.37 (br s, 1H, OH), 4.56 (br s, 2H, 2 × OH), 4.73 (symm m, 1H, furan-CH(OH)), 5.09 (br s, 1H, OH), 6.41 (d, *J* = 0.6 Hz, 1H, furan-H), 12.42 (br s, 1H, COOH); ^13^C NMR (100 MHz, DMSO-*d_6_*) δ (ppm) = 13.37, 63.25, 65.83, 70.92, 72.47, 107.00, 114.03, 155.06, 156.59, 164.90; IR (ATR) υ = 3281, 2903, 1672, 1581, 1441, 1238, 1084, 1037, 944, 877, 851, 631, 568 cm^–1^; HRMS (ESI) m/z calculated for C_10_H_13_O_7_ [M–H]^–^ 245.0661, found 245.0666; purity by HPLC: 99.10%.

### NMR characterization of 2-((4-Methoxyphenyl)amino)benzoic acid (6G7)

Light green solid. ^1^H NMR (400 MHz, DMSO-*d_6_*) δ (ppm) = 3.76 (s, 3H, OCH_3_), 6.68 (ddd, *J*_1_ = 8.0 Hz, *J*_2_ = 7.1 Hz, *J*_3_ = 1.0 Hz, 1H, Ar-H), 6.92 (dd, *J*_1_ = 8.4 Hz, *J*_2_ = 1.0 Hz, 1H, Ar-H), 6.94-6.98 (m, 2H, Ar-H), 7.16-7.20 (m, 2H, Ar-H), 7.32 (ddd, *J*_1_ = 8.4 Hz, *J*_2_ = 7.1 Hz, *J*_3_ = 1.8 Hz, 1H, Ar-H), 7.86 (dd, *J*_1_ = 8.0 Hz, *J*_2_ = 1.8 Hz, 1H, Ar-H), 9.43 (br s, 1H, NH), 12.96 (br s, 1H, COOH); ^13^C NMR (100 MHz, DMSO-*d_6_*) δ (ppm) = 55.19, 111.14, 112.70, 114.70, 116.22, 125.04, 131.72, 132.84, 134.18, 148.75, 156.02, 170.01; IR (ATR) υ = 2997, 2953, 2836, 2640, 2567, 1658, 1597, 1575, 1509, 1451, 1441, 1423, 1232, 1181, 1171, 1110, 1029, 909, 841, 822, 752 cm^–1^; HRMS (ESI) m/z calculated for C_14_H_14_NO_3_ [M+H]^+^ 244.0974, found 244.0980; purity by HPLC: 99.39%.

### NMR characterization of 2-(2-(1*H*-indol-3-yl)acetamido)phenethyl 2-(1*H*-indol-3-yl)acetate (7C8)

Off-white solid. ^1^H NMR (400 MHz, DMSO-*d_6_*) δ (ppm) = 2.80 (t, *J* = 7.0 Hz, 2H, OCH_2_CH_2_), 3.66 (s, 2H, CH_2_COO), 3.75 (s, 2H, CH_2_CONH), 4.06 (t, *J* = 7.0 Hz, 2H, OCH_2_CH_2_), 6.95 (ddd, *J*_1_ = 9.4 Hz, *J*_2_ = 6.9 Hz, *J*_3_ = 1.1 Hz, 1H, Ar-H), 6.98 (ddd, *J*_1_ = 9.2 Hz, *J*_2_ = 7.2 Hz, *J*_3_ = 1.0 Hz, 1H, Ar-H), 7.03-7.10 (m, 3H, Ar-H), 7.13 (dd, *J*_1_ = 7.9 Hz, *J*_2_ = 1.5 Hz, 1H, Ar-H), 7.16-7.21 (m, 2H, Ar-H), 7.27 (d, *J* = 2.1 Hz, 1H, Ar-H), 7.32-7.37 (m, 3H, Ar-H), 7.41 (app d, *J* = 7.9 Hz, 1H, Ar-H), 7.62 (app d, *J* = 8.2 Hz, 1H, Ar-H), 9.39 (br s, 1H, NHCO), 10.93 (br s, 2H, 2 × indole NH); ^13^C NMR (100 MHz, DMSO-*d_6_*) δ (ppm) = 29.99, 30.62, 33.02, 63.56, 106.82, 108.52, 111.31 (2C), 118.34, 118.42, 118.43, 118.51, 120.98 (2C), 123.93, 124.01, 125.42, 126.01, 126.70, 126.98, 127.12, 130.04, 132.15, 136.00, 136.10, 136.30, 170.01, 171.40; IR (ATR) υ = 3370, 3056, 1721, 1658, 1586, 1509, 1455, 1338, 1297, 1246, 1156, 1123, 1094, 1065, 1008, 739, 675 cm^–1^; HRMS (ESI) m/z calculated for C_28_H_24_N_3_O_3_ [M–H]^–^ 450.1818, found 450.1829; purity by HPLC: 96.98%.
